# Nutrient consumption and associated factors among school age children in Dewa Chefe District, northeast Ethiopia: a cross-sectional study

**DOI:** 10.1186/s13104-018-3773-z

**Published:** 2018-09-17

**Authors:** Endris Seid, Lemma Derseh, Terefe Derso, Mekonnen Assefa, Kedir Abdela Gonete, Amare Tariku

**Affiliations:** 1 Department of Human Nutrition, Institute of Public Health, College of Medicine and Health Sciences, University of Gondar, Gondar, Ethiopia; 2 Department of Epidemiology and Biostatistics, Institute of Public Health, College of Medicine and Health Sciences, University of Gondar, Gondar, Ethiopia; 3 Department of Social and Public Health, College of Medicine and Health Sciences, Debre Tabor University, Debre Tabor, Ethiopia

**Keywords:** Nutrient adequacy, School aged children, Northeast Ethiopia

## Abstract

**Background:**

Inadequate nutrient consumption causes protein energy malnutrition and micronutrient deficiencies and related consequences, including poor physical growth and intellectual development. However, literatures showing quantitative measurement of dietary intake of children are limited in Ethiopia. Therefore, this study investigated nutrient consumption and associated factors among school age children (7–9 years) in Dewa Cheffe District, northeast of Ethiopia.

**Methods:**

A community based cross-sectional study was conducted from November to December, 2015 in Dewa Cheffe District. A multistage sampling technique was used to select 605 study subjects. Pre-tested and structured questionnaire was used to collect data. A 24-h dietary recall with portion size estimation method was used to assess nutrient consumption of school age children. Multivariable logistic regression analysis was fitted to identify factors associated with inadequate energy intake. adjusted odds ratio (AOR) with corresponding 95% confidence interval was computed to show the strength of association. In multivariable analysis, a *P* value of < 0.05 was used to declare statistically significance.

**Results:**

A total of 600 school age children were included in the study. About 29% [95%, CI 21.9, 36.1] of study participants had inadequate energy intake. The result of multivariable analysis revealed that, children who were belonged to a female headed households [AOR = 3.65; 95% CI 1.20, 11.04] and family size of six and above [AOR = 14.42; 95% CI 4.65, 44.67] were found with increased odds of inadequate energy consumption. In contrast, decreased odds of inadequate energy consumption were observed among children whose mothers were housewives [AOR = 0.32; 95% CI 0.11, 0.52].

**Conclusions:**

In this study, one-third of school children had inadequate energy consumption. Female headed households, being in the larger family size and housewives mother were significantly associated with inadequate energy consumption. Therefore, giving special focus to female headed households, large family and outdoor worker mothers will help to improve dietary intake of children.

**Electronic supplementary material:**

The online version of this article (10.1186/s13104-018-3773-z) contains supplementary material, which is available to authorized users.

## Background

Children are building blocks of the future generation [1] and their health, physical growth, and intellectual development fundamentally depends on adequate provision of nutrients [1–4]. Researchers also showed that school performance of children depends on their optimal nutritional status [3, 4]. On the other hand, inadequate nutrient consumption is strongly associated with protein energy malnutrition and micronutrient deficiencies [5, 6]. About 20–80% of school children in developing countries are suffering from poor nutrient consumption and undernutrition [7, 8]. In Ethiopia school-age children are affected by a wide range of nutrition related problems, mainly due to inadequate food consumption, which constrains their ability to thrive and benefit from education [9–13]. Undernutrition is contributed to 28% of child mortality, loss of cognitive performance, higher medical expenses and loss of 55.5 billion birr per annum [14, 15].

With due cognizant of the adverse consequences of undernutrition, the country designed and has been implemented different nutritional intervention with the major objective of ensuring optimal feeding practices so as to address the higher magnitude of undernutrition thereby mortality [16, 17]. However, the recent National Food Consumption Survey revealed that nutrient inadequacy was a major health concern and the average total calorie intake of children was below recommended intakes in all regions of the country [18]. Likewise, the study reported from the Western part Ethiopia, Nekemtie District, demonstrated that about 76.42% of school age children had inadequate energy intake, and the consumption of other nutrients was also found as less than the recommended dietary allowance (RDA) [19].

The result of different studies conducted elsewhere noted that inadequate energy and nutrient intake was associated with various factors, but mainly related to the socio-demographic and economic characteristics. Accordingly, low socioeconomic status [20–22], unemployment [20] and illiteracy status of the mothers [20–23], large family size [20], food insecurity [24], nuclear families type [20] and sex of children [7, 20, 25] are factors which negatively influences adequate nutrient consumption of children.

Mostly undernutrition like stunting at school age is associated with poor nutrition since early childhood and the degree of stunting tends to increase throughout the school-age years. However, children can exhibit catch-up growth if their environment improves. Therefore, interventions in school-age children can supplement efforts in preschool years to reduce levels of stunting and related effects on children’s health and education [26]. Though evidences showing the dietary consumption of school children have a decisive role to tackle the adverse and functional consequences of poor dietary intake, literatures are scarce in Ethiopia. Hence, this study estimated inadequate energy intake and associated factors among school age children (7–9 years) in Dewa Cheffe District, northeast Ethiopia.

## Methods

### Study setting and design

A community based cross- sectional study was conducted from November to December, 2015 in Dewa Cheffe District. Dewa Cheffe is one of the seven districts of Oromia Zone in Amhara Regional State. The district is found 325 km away from Addis Ababa, the capital of Ethiopia. The district is divided into 21 Kebeles (*the smallest administration unit*). Based on the 2015/2016 the district Finance and Economic Development office projection, the total population is estimated at 141,529, and school children constitute 15.7% (22, 176) of the total. Livestock and crop productions are important sources of livelihoods in the district. Sorghum, Teff, Maize, Barley and Wheat are the main crops cultivated in the setting.

### Sample size and sampling procedure

A randomly selected school aged children (7–9 years) living in Dewa Chefe District were included in the study, however critically ill and those with already established Diabetes Mellitus were excluded from the study. The required sample size was determined by using single population proportion by taking into account of the following assumptions: expected prevalence of adequate energy consumption as 50% (since there was no prior study in the area), 95% level of confidence and 5% margin of error. In addition, a 5% non-response rate and design effect of 1.5 was anticipated to obtain the final sample size of 605.

A multi-stage stratified sampling followed by a systematic random sampling technique was employed to reach the study subjects. The kebeles were stratified into urban and rural kebeles. Of the total twenty-one kebeles, five (one urban and four rural) were selected by lottery method. The total number of households with school age children was accessed from the District Administration Office. The numbers of households with eligible children in the selected kebeles were determined proportionally. In addition, the sampling interval was calculated, and households with eligible children were selected using a systematic random sampling technique. For households with more than one study subject, only one was selected using lottery method. When mother–child pairs were not available at the time of data collection, two repeated visits were made.

### Data collection procedures

Data was collected using pretested, structured and interviewer administered questionnaire consisted of socio-demographic and dietary related information. Dietary intake information was collected using a semi-quantitative food frequency questionnaire (FFQ) developed based on internationally recognized multiple pass method described by Gibson and Ferguson [27]. Initially, English version questionnaire was translated into Amharic and then back translated to English by language and Public Health experts to maintain its consistency. Six clinical nurses and two Health Officers were recruited as data collector and supervisor, respectively. Two days training was given for data collectors and supervisors. Prior to data collection, the questionnaire was pretested on 5% (30) of respondents in one Kebele which was not included in the sample but with similar setting to evaluated acceptability and applicability of procedures and tools.

### Measurement of nutrient intake

Nutrient intake of children was collected using 24-hour recall method. Each interview considered a stepwise series of questions. In addition, commonly used household utensils, food substitutes and pictures of most frequently consumed foods in the study area were used to improve accuracy of dietary measurement and minimize recall bias. As recommended by Gibson and Ferguson [27], the dietary interviews for this survey were structured in “Four Passes Method” to enhance participants memory. In the first place mothers were asked to report all food items consumed by the child in the previous 24 h. Secondly, portion size of food was estimated and then in case of mixed dish detail information regarding the ingredients of food was obtained. Finally, verification of the collected dietary information was done. The average portion size for each type of food was determined using commonly used utensils. A digital food scale was used to measure the amount of food consumed and of ingredients used in food preparation. Moreover, the method of preparation was also considered. After calculating the child’s net consumption, the energy and nutrient contents or value of the food item was determined using the Ethiopian Food Composition Table Part IV [28]. The data were subsequently converted into the amount of energy and nutrient intake per individual per day. Finally, the nutrient consumption was compared with the estimated average requirements or Intake (EAR) or recommended dietary allowance (RDA) for National Institute of Nutrition (ICMR, 2004). Nutrient adequacy was expressed as ‘percent of adequacy’ by comparing nutrient intake of the children to RDA. ‘Adequate energy intake’ was ascertained if a child had nutrient intake of greater than or equal to estimated average requirements (EAR), whereas ‘inadequate energy intake’ was defined as nutrient intake of lower than EAR. Nutrient intake greater than or equal to Recommended Daily Allowance (RDA) was considered as ‘Adequate intake’ while nutrient intake below the RDA was classified as ‘Inadequate Intake’. Dietary diversity was estimated through classifying the reported food items into seven food groups, as starchy staples (grains, roots, and tubers); legumes, nuts and seeds; vitamin-A rich fruits and vegetables; other fruits and vegetables; egg; dairy products (milk, yoghurt, and cheese); and flesh foods (meat, fish, poultry, and organ meats) [29]. Furthermore, mothers were requested to estimate the frequency of consumption of the above food groups.

Household wealth index was computed using a composite indicator for urban and rural residents by considering the following properties; selected household assets, and size of agricultural land. Principal component analysis was performed to categorize the household wealth index into lowest, middle and highest.

### Data management and analysis

Data were entered into Epi-info version 3.5.1 and exported to SPSS version 20 for analysis. Descriptive statistics using frequencies and proportions were used to summarize variables. Binary logistic regression model was fitted. Variables with P-value < 0.2 in bivariable analysis were entered into multivariable analysis. Both Crude Odds Ratio (COR) and adjusted odds ratio (AOR) with corresponding 95% confidence interval (CI) were computed to show the strength of association. In multivariable analysis, variables with P-value < 0.05 were considered as statistically significant.

## Results

### Socio-demographic and economic characteristics

A total of 600 mother–child pairs were interviewed with the response rate of 99.2%. About 60.2% of children were males. Majority (89.7%) of the households were headed by men. Moreover, about two-third (65.7% and 57.8%, respectively) of mothers were housewives and illiterate (Table 1).

**Table 1:** Socio-demographic and economic characteristics of mother–child pairs in Dewa Cheffe District, northeast Ethiopia, 2015 (n = 600)

Variables	Frequency	Percent
Sex of the child		
Male	361	60.2
Female	239	39.8
Age of child (years)		
7	193	32.2
8	184	30.7
9	223	37.2
Head of household		
Male	538	89.7
Female	62	10.3
Age of the mothers		
< 25 years	47	7.8
26–35 years	273	45.5
36–45 years	201	33.5
≥ 46 years	79	13.2
Mother’s education		
Illiterate	347	57.8
Can read and write	207	34.5
Primary school and above	46	7.7
Mother’s employment status		
Housewife	394	65.7
Self-employed	206	34.3
Father’s education		
Illiterate	319	53.2
Can read and write	264	44.0
Primary school and above	17	2.8
Wealth status		
Lowest	150	25.0
Medium	301	50.2
Highest	149	24.8
Family size		
≤ 3	110	18.3
4–5	232	38.7
≥ 6	258	43.0

### Dietary diversity, nutrient and energy intake of children

**Fig. 1:**
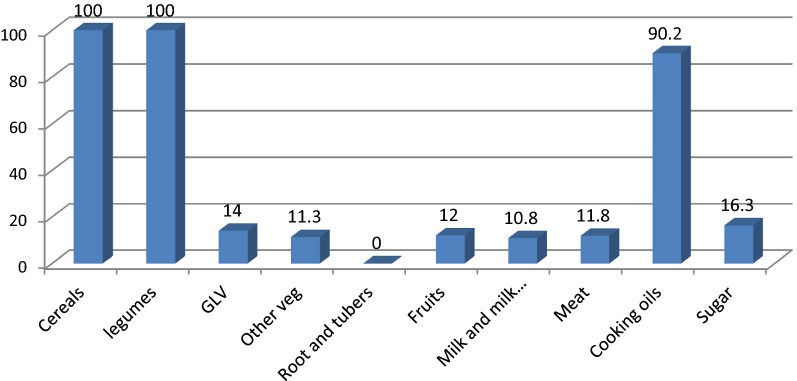
Dietary diversity of school aged children in Dewa Cheffe District, northeast Ethiopia, 2015 (n = 600)

The dietary habits of children were mainly based on cereals (100%) and legumes (100%). Only few of participants consumed milk products (10.8%), meat (11.8%), and green leafy vegetables (GLV) (14%). Furthermore, none of the children ate roots and tubers (Fig. 1). Almost all (98%) and three-fourth (75%) of children consumed sugar and fruits fortnightly, respectively. However, about one-quarter (25%) and one-third (32.2%) of children didn’t consume milk and GLV over the past 1 month prior to the date of survey, respectively (Table 2).

**Table 2:** Frequency of food consumption of foods from food groups among school age children across the reference periods preceding the date of survey in Dewa Cheffe District, northeast Ethiopia, 2015 (n = 600)

Food groups	Every day (%)	Once week (%)	Fortnightly (%)	Once in month (%)	Never (%)
Cereals	100	0	0	0	0
Pulse/legumes	100	0	0	0	0
Green leafy vegetables	0	22.7	45	0.2	32.2
Roots and tubers	0	32	43	25	0
Fruits	0	0	75	25	0
Milk and milk products	0	0	16.5	58.5	25
Meat	0	16.5	32	51.5	0
Cooking oils	100	0	0	0	0
Sugar	0	0	98	0	2

Almost all of children took protein (99.0%) and iron (98.5%) at the amount greater than or equal to their RDA. However, only half of the study participants were found with adequate intake of Calcium (56.0%), Niacin (50.7%) and β-carotene (44.5%) (Table 3).

**Table 3:** The mean daily nutrient intake of school age children in Dewa Cheffe District, Northeast Ethiopia, 2015 (n = 600)

Nutrients	Mean nutrient intake compared to RDA		% of children consumed nutrients ≥ EAR or RDA
	RDA	Mean nutrient intake	
Energy (kcal)	1690	1371.56 ± 172.47	71^a^
Protein (g)	29.5	29.21 ± 6.51	99.0^b^
Calcium (mg)	600	336.39 ± 90.56	56.0^b^
Iron (mg)	16	15.76 ± 2.76	98.5^b^
Vitamin C (mg)	40	14.14 ± 8.25	35.4^b^
β-carotene (micro gram)	4800	213.6 ± 96.19	44.5^b^
Thiamine (mg)	0.8	0.5 ± 0.13	62.5^b^
Riboflavin (mg)	1.0	0.64 ± 0.4	64.0^b^
Niacin (mg)	13	6.59 ± 0.91	50.7^b^

^a^EAR, ^b^ RDA

In this community, there was no statistical difference in the mean energy consumption among males (1375.81 ± 211.82) and females (1366.62 ± 294.51). But, mean protein (32.32 gm versus 25.58 gm) and Calcium (381.54 mg versus 270.81 mg) intake was significantly higher in males than females at a P-value of < 0.05 (Table 4). Concerning energy consumption, about 29.0% [95%, CI 21.9, 36.1] of children had inadequate energy intake (took < of their EAR), whereas 71% of children had adequate energy consumption (took ≥ of their EAR).

**Table 4:** Mean nutrient intake among school age children by sex in Dewa Cheffe District, northeast Ethiopia, 2015

Nutrients	RDA	Mean nutrient intake (n = 600)	Male Mean nutrient intake (n = 323)	Female Mean nutrient intake (n = 277)	P-value
Energy (kcal)	1690	1371.56 ± 172.47	1375.81 ± 211.82	1366.62 ± 294.51	0.208
Protein (g)	29.5	29.21 ± 6.51	32.32 ± 5.96	25.58 ± 10.74	0.000
Calcium (mg)	600	336.39 ± 90.56	381.54 ± 143.25	270.81 ± 58.76	0.000
Iron (mg)	16	15.76 ± 2.76	16.39 ± 6.32	15.02 ± 10.2	0.08
Vitamin C (mg)	40	14.14 ± 8.25	14.71 ± 5.20	13.48 ± 10.76	0.27
β carotene (micro gram)	4800	213.6 ± 96.19	213.6 ± 62.81	208.6 ± 71.35	0.37
Thiamine (mg)	0.8	0.5 ± 0.13	0.67 ± 0.21	0.61 ± 0.27	0.41
Riboflavin (mg)	1.0	0.64 ± 0.4	0.05 ± 0.21	0.14 ± 0.3	0.50
Niacin (mg)	13	6.59 ± 0.91	7.13 ± 1.2	6.83 ± 1.74	0.42

### Factors associated with inadequacy energy consumption

Both bivariate and multivariate logistic regressions were done; the result of multivariate analysis revealed that, children who were belonged from households headed by females [AOR = 3.65; 95% CI 1.20, 11.04] and family size of six and above [AOR = 14.42; 95% CI 4.65, 44.67] were found with increased odds of inadequate energy consumption. In contrast, decreased odds of inadequate energy consumption was observed among children whose mothers were housewives [AOR = 0.32; 95% CI 0.11, 0.52] (Table 5).

**Table 5:** Factors associated with inadequacy energy consumption among school age children in Dewa Cheffe District, northeast Ethiopia, 2015 (n = 600)

Variables	Adequacy of energy intake		COR (95% CI)^a^	AOR (95% CI)^b^
	Inadequate	Adequate		
Age of child (in years)				
7	69	124	1	
8	44	140	0.57 (0.36, 0.89)*	
9	61	162	0.68 (0.45, 0.93)*	
Head of household				
Male	134	404	1	1
Female	40	22	5.482 (3.15, 9.56)*	3.65 (1.20, 11.04)*
Mother’s education				
Illiterate	154	193	4.45 (1.94, 10.22)*	
Can read and write	13	194	0.37 (0.14, 0.99)	
Primary school and above	7	39	1	
Mother’s occupation				
Housewife	160	234	0.10 (0.06, 0.19)*	0.32 (0.11, 0.52)*
Self employed	192	14	1	1
Family size				
≤ 3	9	118	1	1
4–5	14	222	0.83 (0.35, 0.97)*	0.85 (0.93, 10.28)
≥ 5	151	86	23.02 (11.12, 47.66)*	14.42 (4.65, 44.67)*
Mother’s age				
≤ 25	7	40	0.17 (0.07, 0.43)*	
26–35	75	198	0.37 (0. 22, 0.62)*	
36–45	52	149	0.34 (0.2, 0.59)*	
≥ 46	40	39	1	

* p < 0.05

^a^Crude odds ratio

^b^Adjusted odds ratio

## Discussions

The present study revealed that, about 29.0% of children had inadequate energy intake which slightly higher than the finding from Kenya (17.3%) [30]. However, the result is lower than the previous studies in western Ethiopia (76.42%) [19] and India (62%) [20]. The observed discrepancy may be due to the difference in the study setting, in which the current study was conducted in the rural area where most of the residents support their livelihood through agricultural production of crops. In addition, the difference could be related to seasonal variation in market availability of food. In Ethiopia, from November to December (study period) agricultural products (crops) were harvested and more available and affordable in the market which in turn positively affects the household’s per capita food availability and food intake. However, the current finding was slightly higher than the finding from Kenya (17.3%) [30].

Besides, the study revealed significant difference in energy consumption with sex of the children which was consistent with former report in the rural Bangladesh [31]. Obliviously, there is cultural bias favoring males in Ethiopia and other developing countries. Children are considered as important source of future labor on the family farm, and expected to provide economic and social security for their parents when their father is debilitated or get old or in times of distress. Consequently, males are prioritized to by parents while serving food, including the nutritious one.

The result of the adjusted analysis revealed that, the likelihood of inadequate energy consumption was higher among children who lived in households headed females and larger family size. However, decreased odds of inadequate energy consumption were common among children of housewife mothers as compared to children of the self- employed mothers. Similar findings were reported in India [20] and Ghana [23]. This might be due to housewife mothers had better time to care and feed their child as compared to outdoor worker mothers.

Being in the large family size (≥ 5) was associated with increased odds of inadequate energy consumption among school aged children compared to their counter parts who belonged from smaller family size (≤ 3). Similar finding was reported in India [20]. Large family poses additional challenges to the household to secure the per capita food availability. The condition worsens in the countries where unemployment rate is high which increases the socio-economic dependency of the household members in the country at large [14].

The odds of in adequate energy consumption were higher among children living under households headed females. This could be explained by in most of developing countries literacy rate was low among women’s, as a result they were engaged in non-professional and low income generating activities. Furthermore, they are also responsible to take care their child and the household members at large which ultimately decreases the time spent to work. This all conditions negatively affects their income and the amount of money spent for purchasing food as compared to their male counterparts.

This study quantitatively estimated nutrient and energy consumption of children. Also, a multiple pass recall method was used to enhance memory of respondents. Despite these methodological strengths, the study is not free from recall bias while qualitatively estimating dietary consumption of children.

## Conclusions

This study showed that nutrient and energy inadequacy was high among children in Dawa Chefe District. Moreover, sex of household head, family size and occupational status of the mother were significantly associated with energy consumption of children. Therefore, attention has to be paid on households headed by female, outdoor worker mothers and large families to improve energy consumption of children. Also, current efforts focused on enhancing micronutrient intake of children should be strengthened.

## Additional file

**Additional file 1.** Questionaire.

## Abbreviations

AOR: adjusted odds ratio
CI: confidence interval
COR: crude odds ratio
EAR: estimated average requirements
FFQ: Food Frequency Questionnaire
RDA: recommended dietary allowance
GLV: green leafy vegetables
SD: standard deviation
