# Supplementary material for: Nutrient consumption and associated factors among school age children in Dewa Chefe District, northeast Ethiopia: a cross-sectional study
Source: BMC Res Notes. 2018 Sep 17;11:669. doi: 10.1186/s13104-018-3773-z (PMC6142308; doi:10.1186/s13104-018-3773-z)
Supplement: Supplementary file 1 — Additional file 1. Questionaire. [file 13104_2018_3773_MOESM1_ESM.docx]

**Annex Seven: Engish Version of Questionnaire**

**1. Household Questionnaire**

**Part I: Participant identification and demographic questions**

Questionnaire number_______________

Date: _____/_____/_________

Participant code ___________ Name of Kebele___________

**II .General information of the household**

1. Respondent 1.Mother 2. Female 3.Caregiver of the child

2. Language of the respondent 1.Amharic 2. Ormigna 3. Tigrigna 4. Other (specify) __

3. Religion of household. 1. Muslim 2. Orthodox Christian 3. Other (Primary school, cycle 1 (grade 1-4)

4**.** Relationship to study child 1. Mother 2. Other female caregiver 3. Father 4. Male caregiver

5. Sex of head of household 1=male, 2=female

**III .Education & occupation status of mother/caregiver**

6. What level of schooling does the child mother**/**caregiver have?

1 = None /illiterate, 2= can read and write, 3 = Primary school, cycle 1 (grade 1- 4), 4= Primary school, cycle 2 (grade 5-8), 6= high school, 5= other (specify) ___

7. Did you contribute to household income during last 12 months? 1= yes, 2= no, 99= Unknown

8. What is the occupation of the child’s mother/caregiver? 1=Unpaid family/household worker, 2=Self-employed (informal), 3 = Self-employed (formal), 4 = Private organization employee, 5 = government employee, 6= 0ther (specify) ______

**IV. Education & occupation status of father**

9. What is his primary occupation? 1= farmer, 2= Handcraft, 3. =Local merchant, 4 =Civil servant, 5= Daily laborer, 6= 0ther (specify) ------

10. What level of schooling does the child’s father have?

1=none /illiterate, 2= can read and write, 3=Primary school, cycle 1 (grade 1-4), 4= Primary school, cycle 2 (grade 5-8), 6= high school, 5= other (specify) _

**V. Child of (7-9 years of age) –study participant**

11. Sex of study child 1. Male 2. Female

12. Age of study child ____ years

13. Have you attended your education in the last 2007 academic year in Ethiopian calendar? 1= Yes, 2= No

14. What is your level of education? Grade completed-----

**VI.HOUSEHOLD INFORMATION**

15. What is your major source of drinking water for members of your household? =.Tap/ piped, 2= protected well/spring, 3= unprotected well/spring, 4=river/lake/pond, 5= other (specify) ___

16. How far is it to your source of drinking water? 1 = in the house, 2=less than 15 minutes, 3=15-30 minutes, 4=30-60 minutes, 5= more than 1 hour, 6=other: ______________ 9=unknown

17. What kind of toilet do you use? 1= field outside, 2= pit latrine, 3= other (specify) ___

18. What is the nearest health facility for your household? 1= Clinic, 2= health center, 3= health station, 4= hospital

19. How far is the nearest health facility? 1= less than hour, 2=more than hour

20. What do you use as a source of energy for cooking in your household? 1=wood, 2= animal dung, 3=charcoal, 4=gas, 5= other (specify) ___

21. Do you cook in the same room as your living quarters? 1= yes, 2= no

22. Do you have a separate kitchen? 1= Yes, 2= No

23. If yes to 22, 1= it is attached, 2= detached from main house

24. Where are the cattle kept? 1= same dwelling, 2= in the barn, 3=outside field 4= unknown, 5= not applicable

**VII. Socioeconomic characteristics of the household**

25. By what means did you get food for the household this past month? (Three most important)

1=Own agricultural production, 2= Daily laborer, 3= small animal husbandry/chicken, 4=from friends, 5=from family, 6=from food relief, 7=from food for work/ food security program , 8= gathered wild food , 9=commerce ,10= other (specify) ___

26. If food for work/ food security program, how often is obtained? 1= weekly, 2= monthly, 3= quarterly, 4= bi-annually, 5= annually, 6= other ( specify)____________

27. If agriculture , how many different fields does the household cultivate and harvest? ------number of fields

28. Do you own the following farm equipment? 1= yes .2= no

28(a).Plough ---------

28(b) Cultivator---------

28(c) Cart--------

28(d) Wheelbarrow-------

28(e) Hoe/shovel-------

28(f) Machete/scythe-------

28(g) Tractor--------

28(h) Other ( specify)-------

1. Do you cultivate the following crops? 1=Yes , 2= No

29(a) Sorghum

29(b) Millet

29(c) Barely

29(d) Tef

29(f) Maize

29(g) Wheat

29(h) Lentils

29(i) Oil crops

29(j) Beans

29(k) Peas

29(l) Chickpeas

29(m) other (specify) ------

30. What are the two most important crops you cultivate? (a) Type of crop and number of bags----------------------

(b) a) type of crop and number of bags------------------

31. Do you have irrigation farm land? 1= yes 2= no

32. If question 31 is yes, what do you usually grow?

1---------, 2------------,3---------- 4-----

33. How many of each type of animals are owned by the household? (Enter 0 for none or actual number of individuals, unknown enter 99)

1. (a) Oxen---------

33(b) Cow---------

33(c) Sheep------

33(d) Goats-------

33(e) Camels----

33(f) Horses----

33(g) Donkeys----

33(h) Mules----

33(i) poultry----

33(j) Other (specify)-------

34. Do you own this residence? 1=yes, 2=No

35. Does any member of the household own the following items ,in good working condition 1= yes , 2= No

35(a) Sewing machine that works ____________

35(b) Bicycle that works_______________

35(c) Radio that works__________

35(d) Tape that works__________

35(e) Television that works________

35(f) Cellular phone that works_______

36. Does any member of this household work on someone else’s land fulltime, part-time, seasonally 1=yes, 2=No

37. If yes Q to #36, how many household members work on someone else’s land fulltime, part-time, seasonally?

37(a) Fulltime-------------

37(b) Part-time-----------

37(c) Seasonally---------

**VIII. Description of housing**

Interviewer, for the following questions, please, ascertain by observing the housing characteristics during the interview process.

38. How many rooms do you have in the house? 1= One, 2= Two, 3= Three, 4= Four, 5= Five or more

39.Do you have a separate kitchen? 1= Yes, 2= No

If yes to, 1= it is attached, 2= detached from main house

40. What is the main material of the floor? 1=dirt, 2= mud, 3= plastic, 4=wood, 5= cement/cement brick, 6=stones, 7= wall paper, 8= other (specify) ---------- 9= Unknown

41. What is the main material used for wall construction?

1=Wood and mud, 2=Wood and thatch, 3=Stone and mud, 4=Stone and cement, 5= other (specify)--------

42. What is the main material used for roof? 1= Thatch, 2= Corrugated iron sheet 3=plastic, 4=wood, 5= cement/cement brick, 6= other (specify) ------------

43. What is the main material used for ceiling?

1= Has no ceiling, 2= Wood, 3= other (specify) -------------

44. Do you have the following commodities? 1= Yes, 2= No

(a). Tables----------

(b). Chairs-----

(C). Bed----------

**IX. Household size**

45. How many individuals live together in this household? (Enter 0 for none or actual number of individuals, unknown enters 99)?

1= 0-6yrs -----------, 2= 7-9 yrs------------, 3=10-18 yrs-------,4=19-49------yrs, 5=50>yrs-----

**X. Food type and sources**

46. What was your main staple food *in the past 4 weeks*? (***2 most important****)*

1= Sorghum, 2= Barley, 3= Teff, 4= Maize, 5= Wheat, 6= Millet, 7= Haricot bean, 8= Milk, 9= Sweet potato, 10= Irish potato, 11= Cassava, 12= Yam 13= Enset, 14=Rice,

15= other - *specify*______________

47. What were the most important sources of your main staple food *in the past 4 weeks*? *(****2 most important****)*

1= Own production 2= Purchase,3= Emergency food /GFD/ 4= Labour exchange through grain,

48. What will be the most important source of your main staple food in the *next 3 months? (****2 most important****)*

1= Own production 2= Purchase, 3= Emergency food /GFD/ 4= Labour exchange through grain,

5= Borrowing (grain), 6= Remittance (grain), 7= Safety Net, 8= Other - *specify*___________________

**XI. Sources of cash income**

49. What is your normal source of cash income *at this time of the year*? *(****3 most in order of importance****)*

1= sale of red pepper 2= Sell of livestock, 3= Sell Of own crop product, 4= Sell of animal product,

5= Sell of fire wood/charcoal, 6= Petty trade, 7= Sell of labour 8= Credit (gov.) 9= Remittance (cash), 10= Sell of fodder/straw/grass, 11= Sale of homemade hand Crafts, 12= sale of fruit and vegetables,

13= Safety Net, 14= Hair dressing, 15= Weaving, 16= rent pack animals, 17= Borrowing,

18= Sale of chat, 19= sale of coffee, 20= sale of Enset/Bula, 21= Used saved money, 22=

50.What is your main source of cash *in the past three months*? *(****3 most in order of importance****)*

1= sale of red pepper 2= Sell of livestock, 3= Sell Of own crop product, 4= Sell of animal product, 5= Sell of fire wood/charcoal, 6= Petty trade, 7= Sell of labour 8= Credit (gov.) 9= Remittance (cash),10= Sell of fodder/straw/grass, 11= Sale of homemade hand Crafts, 12= sale of fruit and vegetables,

13= Safety Net, 14= Hair dressing, 15= Weaving, 16= rent pack animals, 17= Borrowing, 18= Sale of chat, 19= sale of coffee, 20= sale of Enset/Bula, 21= Used saved money, 22= Others______________

**XII. Coping mechanism**

51. What is your coping mechanism at stress time? (whenever they face food shortage)*(****3 most important****)*

1= Sale of productive animals, 2= Sale of more animals, 3= Sale of plough animals (Oxen) 4= Reduce family size, 5= Reduce number of meals/size, 6= Sale of farming tools, 7= Petty trade, 8= Migration for labour/food, 9= Sale charcoal/firewood, 10= Rent pack animals, 11= Remittance, 12= Borrowing, 13= Sale of Relief food, 14= Stress is not experienced, 15= Eating wild food, 16= Safety Net, 17= Consume stored food (seed), 18= Rent farm land, 19= Sale of personal asset (jewellery), 20= Credit from social service (Edir), 21= Credit from church, 22= Sale of labour, 23=other- specify__________________

52.In the past 12 months, did you or your family ever cut the size of your meals or skip meals. Because there wasn’t enough money for food or because you had run out of food stock?0=Yes 1= No

14. If yes question number 12, how often did it happen? 1=. Every month 2= After every three months 3.=Only 1 or 2 months

4 = Do not know.

53. In the last one month, did (child’s name) ever skip meals because there was no food or there was not enough money for food? 1= Yes, = No

If yes above ask, how often this happen? 1=-Almost every day, 2= some days but not every day, 3= in only 1 or 2 days, 4= other, specify--------

**2. 24-hour dietary call -Questionnaire**

1. **First, list all foods and drinks consumed during a 24 hour period.**
   1. List all foods and beverages or drinks consumed by the child. This list can be entered directly into the questionnaire, or onto the separate listing form, and then transferred to the questionnaire.
   2. Identify about what time each food was consumed. For this survey, the reference period is from sunrise yesterday to sunrise this morning:
      1. Morning = 1, about sunrise or time awoke until just before noon meal (12:01-5:30)
      2. Afternoon = 2, noon meal until sunset (~5:30-12:00)
2. Evening/ night = 3, sunset yesterday until sunrise or awaking this morning (12:01-12:00).
3. Second, estimate the amount consumed
   1. Help the mother remember the study child and estimate the amount of each food or drink that her child ate or drank (). Enter these data into questionnaire.
   2. If any food is leftover from what the mother served to the child, enter that amount in into questionnaire If the amount consumed cannot be estimated for the child, you must also:
      1. Enter the total number of individuals who ate together at this meal,
      2. divide them by age/sex categories
      3. enter the amount of this food that was used during this meal, and
      4. Ask food was left over and how much.
4. **Third, get more detail about each food**.
   1. Use standard “probes” (probing questions) to get these details. Details are different for different foods; and for details, describe the following:
      1. What color was it?
      2. What type was it? (this could involve obtaining a recipe for home foods or describing a food that was purchased)
      3. How big was it?
      4. If purchased, what brand was it?
      5. Examples:
         1. If she ate mango, ask if it was ripe or what color inside and the size or weight.
         2. If she ate fish, try to determine what type of fish, and if the whole fish was eaten or just the flesh, if the fish was dried or fresh.
         3. If she made shiro wot at home, ask for the recipe and total amount of shiro wot prepared
   2. Identify the source of the food
   3. Identify how the mother prepared the food (suchas boiled, baked, and fermented). For recipes, inquire whether the ingredient was altered prior to adding it to the dish (such as roasting flour after purchase, spicing butter, etc.). Raw, fermented, fried in oil, etc can change the nutritional content of the food. These are needed to identify the food code for each food or beverage.
5. **Fourth**, **verify everything consumed**.
   1. Quickly read the information back to her and ask her whether anything is missing.
6. List all foods

| Morning | Mid day | Afternoon |
| --- | --- | --- |
|  |  |  |

7.List all foods

| Breakfast | Lunch | Snacks | Dinner |
| --- | --- | --- | --- |
|  |  |  |  |

CONDUCTING THE 24 –HOPUR

| Food item | Meal time | Food description | How was this prepared | Where did you get (food)? | How much did you serve? | If any leftover, how much? |  |  |  |  |
| --- | --- | --- | --- | --- | --- | --- | --- | --- | --- | --- |
|  | Breakfast |  |  |  |  |  |  |  |  |  |
|  | Breakfast |  |  |  |  |  |  |  |  |  |
| Total recipe | Ingredients | Description | Amount | Measure | How prepared | Source | How | Staple foods | Estimate the amount served | leftover or lost |
|  | 1 |  |  |  |  |  |  |  |  |  |
|  | 2 |  |  |  |  |  |  |  |  |  |
|  | 3 |  |  |  |  |  |  |  |  |  |
|  | 4 |  |  |  |  |  |  |  |  |  |
|  | 5 |  |  |  |  |  |  |  |  |  |
|  | 6 |  |  |  |  |  |  |  |  |  |
|  | 7 |  |  |  |  |  |  |  |  |  |
|  | 8 |  |  |  |  |  |  |  |  |  |

| Food item | Meal time | Food description | How was this prepared | Where did you get (food)? | How much did you serve? |  |  |  |  |  |
| --- | --- | --- | --- | --- | --- | --- | --- | --- | --- | --- |
|  | Lunch |  |  |  |  |  |  |  |  |  |
|  | Lunch |  |  |  |  |  |  |  |  |  |
| Total recipe | Ingredients | Description | Amount | Measure | How prepared | Source | How | Staple foods | Estimate the amount served | leftover or lost |
|  | 1 |  |  |  |  |  |  |  |  |  |
|  | 2 |  |  |  |  |  |  |  |  |  |
|  | 3 |  |  |  |  |  |  |  |  |  |
|  | 4 |  |  |  |  |  |  |  |  |  |
|  | 5 |  |  |  |  |  |  |  |  |  |
|  | 6 |  |  |  |  |  |  |  |  |  |
|  | 7 |  |  |  |  |  |  |  |  |  |
|  | 8 |  |  |  |  |  |  |  |  |  |

| Food item | Meal time | Food description | How was this prepared | Where did you get (food)? | How much did you serve? | If any leftover, how much? |  |  |  |  |
| --- | --- | --- | --- | --- | --- | --- | --- | --- | --- | --- |
|  | Snack |  |  |  |  |  |  |  |  |  |
|  | Snack |  |  |  |  |  |  |  |  |  |
| Total recipe | Ingredients | Description | Amount | Measure | How prepared | Source | How | Many people consumed this food at this meal | Estimate the amount served | How much was leftover or lost from this mea; |
|  | 1 |  |  |  |  |  |  |  |  |  |
|  | 2 |  |  |  |  |  |  |  |  |  |
|  | 3 |  |  |  |  |  |  |  |  |  |
|  | 4 |  |  |  |  |  |  |  |  |  |
|  | 5 |  |  |  |  |  |  |  |  |  |
|  | 6 |  |  |  |  |  |  |  |  |  |
|  | 7 |  |  |  |  |  |  |  |  |  |
|  | 8 |  |  |  |  |  |  |  |  |  |

| Food item | Meal time | Food description | How was this prepared | Where did you get (food)? | How much did you serve? | If any leftover, how much? |  |  |  |  |
| --- | --- | --- | --- | --- | --- | --- | --- | --- | --- | --- |
|  | Dinner |  |  |  |  |  |  |  |  |  |
|  | Dinner |  |  |  |  |  |  |  |  |  |
| Total recipe | Ingredients | Description | Amount | Measure | How prepared | Source | How | Many people consumed this food at this meal | Estimate the amount served | How much was leftover or lost from this mea; |
|  | 1 |  |  |  |  |  |  |  |  |  |
|  | 2 |  |  |  |  |  |  |  |  |  |
|  | 3 |  |  |  |  |  |  |  |  |  |
|  | 4 |  |  |  |  |  |  |  |  |  |
|  | 5 |  |  |  |  |  |  |  |  |  |
|  | 6 |  |  |  |  |  |  |  |  |  |
|  | 7 |  |  |  |  |  |  |  |  |  |
|  | 8 |  |  |  |  |  |  |  |  |  |
|  |  |  |  |  |  |  |  |  |  |  |

- Where did you get this food? 1= own production, 2= purchased , 3= gift or food aid
- How was it prepared? 1= raw /no change/as purchased ,2=fermented ,3=fried , 4=cooked or boiled ,5= baked/grilled –dry heat,6= local miller, 7= other ---
- Type of measure: ml=milliliter, L= liter ,3= kg= kilogram , etc.,

1. Children’s food groups recall in the past 24-hour prior to the study

I would like to ask you about liquids or foods that (name of the study child) may have had yesterday during the day or night. I am interested in whether the child had the item even if it was combined with other food?

| **Food groups** | Child’s recall | | |
| --- | --- | --- | --- |
|  | Yes | No | Dk |
|  | 1 | 2 | 3 |
| Bread, rice, wheat, other foods made from cereals/grains? |  |  |  |
| Milk such as animal milk/powdered/tinned? |  |  |  |
| Pulses such as foods made from beans, peas, lentils or nuts? |  |  |  |
| Pumpkin, carrots, squash, sweet potatoes that are yellow or orange? |  |  |  |
| White potatoes, white yams, cassava or any other food made from roots? |  |  |  |
| Any dark green, leafy vegetables? |  |  |  |
| Ripe mangoes, papaya, any other locally available vitamin rich food? |  |  |  |
| Other fruits or vegetables? |  |  |  |
| Liver, kidney , heart ,or other organ meats |  |  |  |
| Any meat such as beef, lamb, goat |  |  |  |
| Eggs |  |  |  |
| Fresh or dried fish or shellfish |  |  |  |
| Cheese, yogurt, other milk product |  |  |  |
| Any sugary foods such as chocolates, sweets, candies, pastries ,biscuits |  |  |  |

4. **Frequency consumption pattern-questionnaire**

In the past one week, the number of times I ate the following food groups, on average every day is about

| Food groups | Daily | | | | | |
| --- | --- | --- | --- | --- | --- | --- |
|  | More than twice | Twice | Once | Less than once | Never | Don’t know |
| Cereals |  |  |  |  |  |  |
| Pulses |  |  |  |  |  |  |
| Green leafy vegetables |  |  |  |  |  |  |
| Other vegetables |  |  |  |  |  |  |
| Roots and tubers |  |  |  |  |  |  |
| Fruits |  |  |  |  |  |  |
| Milk and milk products |  |  |  |  |  |  |
| Meat, fish/egg |  |  |  |  |  |  |
| Fat and oils |  |  |  |  |  |  |
| Sugar and jaggery |  |  |  |  |  |  |
